# Teacher, caregiver, and student acceptability of teachers delivering task-shifted mental health care to students in Darjeeling, India: a mixed methods pilot study

**DOI:** 10.1007/s44192-022-00024-z

**Published:** 2022-10-31

**Authors:** Christina M. Cruz, Choden Dukpa, Juliana L. Vanderburg, Abhishek K. Rauniyar, Priscilla Giri, Surekha Bhattarai, Arpana Thapa, Karen Hampanda, Bradley N. Gaynes, Molly M. Lamb, Michael Matergia

**Affiliations:** 1 Department of Psychiatry, University of North Carolina at Chapel Hill School of Medicine, 101 Manning Drive, CB #7160, Chapel Hill, NC 27599 USA; 2 School Psychology Program, University of North Carolina at Chapel Hill School of Education, Chapel Hill, NC USA; 3 Darjeeling Ladenla Road Prerna, Darjeeling, West Bengal India; 4 Department of Epidemiology, Colorado School of Public Health, Aurora, CO USA; 5 Center for Global Health, Colorado School of Public Health, 131999 E. Montview Blvd., Suite 310, Mail Box A090, Aurora, CO USA; 6 Department of Obstetrics and Gynecology, University of Colorado Anschutz Medical Campus, Aurora, CO USA; 7 Department of Epidemiology, University of North Carolina at Chapel Hill Gillings School of Global Public Health, MacNider Bldg. Suite 304, CB#7160, 333 S. Columbia St., Chapel Hill, NC 27599 USA; 8 Broadleaf Health and Education Alliance, Stroudsburg, PA USA

**Keywords:** Community mental health, Acceptability, Task-shifting, Child mental health care, Global mental health, School mental health, Teacher, Student, Caregiver, Primary school

## Abstract

**Background:**

The acceptability of teachers delivering task-shifted mental health care to their school-aged students is understudied. Here, we evaluate teachers’, students’, and caregivers’ acceptability of *Tealeaf* (Teachers Leading the Frontlines), an alternative system of care in which teachers are trained and supervised to deliver transdiagnostic, non-manualized task-shifted care to their students.

**Methods:**

In a 2019 single-arm, mixed methods, pragmatic acceptability pilot study in Darjeeling, India, 13 teachers delivered task-shifted child mental health care to 26 students in need. Teachers delivered care through using a transdiagnostic, non-manualized therapy modality, “education as mental health therapy” (Ed-MH). Measured with validated scales, teachers’ and students’ acceptability were compared after teacher training (PRE) and at the end of intervention (POST) using paired *t* tests. Teachers (n = 7), students (n = 7), and caregivers (n = 7) completed semi-structured interviews POST.

**Results:**

Teachers’ quantitative measures indicated moderate acceptability PRE and POST and did not change PRE to POST. Children’s measures showed acceptability PRE and POST but decreased PRE to POST. Teachers and caregivers universally expressed acceptability in interviews. Facilitators of acceptability included impact, trust of teachers, and teachers’ ability to make adaptations. Conditions required for acceptability included supervision and teachers emphasizing academics benefits over mental health benefits to caregivers. Barriers to acceptability included a lack of teacher time and stigma. Interviewed students universally were unaware of receiving care; teachers intentionally avoided singling them out.

**Conclusion:**

Teachers, caregivers, and children found teacher delivering task-shifted care acceptable, a key factor in care adoption and sustainability, though interviewed children were unaware of receiving care.

*Trial registration* The trial was registered on January 01, 2018 with Clinical Trials Registry—India (CTRI), Reg. No. CTRI/2018/01/011471, Ref. No. REF/2017/11/015895. http://ctri.nic.in/Clinicaltrials/pdf_generate.php?trialid=21129&EncHid=&modid=&compid=%27,%2721129det%27

**Supplementary Information:**

The online version contains supplementary material available at 10.1007/s44192-022-00024-z.

## Background

The prevalence of youth mental conditions is approximately 10–20% globally [1, 2]. In high income countries (HICs), of youth in need of indicated mental health care, defined as talk therapies and/or medications to treat an identified mental health problem, 20% receive it; access in low and middle income countries (LMICs) tends to be poorer, with 1% or fewer of children in need receiving care [3–5]. Youth with mental health needs remain critically underserved.

A shortage of trained professionals underlies the care gap, necessitating alternative models of care to bridge it, such as task-shifting [6]. In task-shifted mental health care, non-accredited individuals, “lay counselors”, are trained and supervised by professionals to deliver indicated care, typically talk therapies, to those with clinical levels of need. In LMICs, lay counselors delivering task-shifted care have improved mental health outcomes for adults in need of care, with several studies and reviews supporting their effectiveness, including a 2013 Cochrane review updated in 2021 [7–12]. Delivering task-shifted care for children (aged 5–12 years old) has yielded mixed outcomes; community health workers (the most common lay counselor human resource) who have delivered task-shifted child mental health care have not consistently improved children’s symptoms of Post-Traumatic Stress Disorder, Depression, or Anxiety [8]. Such care requires lay counselors to have experience with children’s growing capabilities in cognition and emotion-regulation, often lacking by community health workers [13].

Teachers in LMICs may be positioned to deliver task-shifted child mental health care [13, 14]. They have experience in child development requisite for care delivery, have consistent access to children, and in real time can address children’s mental health needs [13–15]. Teachers in HICs have been studied to deliver indicated mental health care on a limited basis, improving symptoms of children with Conduct Disorder and Attention Deficit Hyperactivity Disorder (ADHD) [16–18]. By contrast, teachers in LMICs have rarely delivered task-shifted care [5, 8, 16]. Rare teacher delivery of indicated care may be due to teachers expressing: (1) being undertrained to work with students with mental health needs; (2) being overburdened with their teaching duties; and (3) that addressing their students’ mental health may not be their responsibility [14].

These factors may underlie whether teachers find it acceptable to deliver task-shifted care to their students. Acceptability is defined as an individual’s judgments of whether a specific model of care is “appropriate, fair, and reasonable” and is considered a key factor in care adoption and sustainability [19–21]. Han and Weiss [22] described 3 domains that affect teacher acceptability of delivering mental health interventions in classrooms, based on work by Elliott [23] and Reimers and colleagues [24]: (1) severity of a student’s target mental health concern; (2) type of care delivered; and (3) required time to deliver care.

The one study that has evaluated teachers’ acceptability of delivering task-shifted care in an LMIC found that teachers considered it acceptable to deliver care to students outside of the classroom setting [25, 26]. No studies have evaluated teacher acceptability of delivering task-shifted mental health care to their elementary school students within classroom settings, where delivering care to their students presents a potential systems-level efficiency given their familiarity with and the consistent time spent with their students [14].

Limited teacher delivery of task-shifted mental health care may also be due to child and family acceptability. To date, however, no published studies have examined the acceptability to children and their families of teacher-delivered, task-shifted mental health care in an LMIC [13]. For adolescents in an LMIC receiving task-shifted mental health care from their teachers, they expressed mixed acceptability of the care [27]. With limited literature on parent acceptability of teacher-delivered care in LMICs, insight may be gained from a relevant HIC study. Parents in this study found it acceptable for teachers to deliver indicated care, citing decreased stigma as compared to outpatient care [28].

To meet the critical global child mental health need with an eye toward implementation outcomes such as acceptability, our group created an alternative system of care called *Tealeaf *[*Tealeaf—Mansik Swastha (Teachers Leading the Frontlines—Mental Health)*] for the Darjeeling region of India*.* In *Tealeaf,* teachers are trained to deliver transdiagnostic, non-manualized, task-shifted care. As *Tealeaf* care is transdiagnostic, it applies “the same underlying principles across mental disorders, without tailoring the protocol to specific diagnoses” [29], thus allowing teachers to learn one set of therapeutic skills for all diagnoses. Care in *Tealeaf* is also non-manualized, allowing teachers to customize the care they deliver, including delivering care within classroom activities or in a more traditional one-on-one (1:1) setting. Early evidence points to teachers delivering *Tealeaf* care feasibly, with fidelity, and with improved mental health symptoms in children who have received *Tealeaf* care [30, 31].

The present study aimed to evaluate, in Darjeeling, India, an LMIC setting, the acceptability to teachers, children, and their families of *Tealeaf.* By studying acceptability, this study may contribute to the literature by evaluating a key prerequisite for sustainable teacher delivery of non-manualized, transdiagnostic, task-shifted child mental health care to their students, which has the potential to help bridge the wide child mental health care gap [3, 4]. Through a 2019 single-arm mixed-methods pilot pragmatic design with parallel data collection and explanatory sequential analysis (QUAN qual), we evaluated whether teachers would find delivering task-shifted mental health care to their students acceptable on top of and integrated into their regular school responsibilities and whether children and their caregivers would find receiving teacher-delivered, task-shifted mental health care in a classroom setting acceptable [32]. We hypothesized that teachers, children, and their families would find teacher-delivered, task-shifted care, i.e., *Tealeaf,* acceptable.

## Methods

### Setting

The study was conducted in Darjeeling, West Bengal, India. The local population is predominantly ethnically Nepali, a minority group in India, and speaks Nepali primarily [33]. A majority of residents work in tea-plantation areas and small-scale agricultural communities, with daily average wages at 176 INR ($2.42 USD) [33]. Despite this economic condition, a majority of rural families with lower incomes prefer sending their children to low cost private (LCP) schools; they perceive LCP schools as providing a high-quality education in English [34]. The number of LCP schools is growing to meet this demand, typically operating without support from the government and leading to enrolled students having poorer access to government services [35]. LCP schools charge a minimal fee and pay their teachers 1500–3000 INR ($23–45 USD) monthly [34]. Child mental health prevalence in Darjeeling is unknown, but a study in a nearby rural area estimated prevalence at 33% [2]. Similarly, access to care in Darjeeling has not been studied; an unpublished needs assessment from our group in 2017 revealed that there were 3 counselors and 1 general psychiatrist available to meet the needs of 100,000 youth. In India more broadly, 1% of children in need receive care [4].

### Participants

Eligible schools were LCP primary schools in the rural areas of the Darjeeling Himalayas and employed at least 3 teachers to allow for a student body size (30–50 students) that was more likely to approximate child psychiatric morbidity estimates [1]. To reach children with the poorest access to care [34], schools were further required to meet the following inclusion criteria: (1) did not receive government aid, (2) charged < 11,500 INR ($180 USD) annual fees, and (3) served families with a daily average income < 725 INR ($10 USD).

To recruit potential schools, we held information sessions for school principals in four communities in rural Darjeeling. Nine of 12 schools attending the information sessions agreed to participate. From participating schools, eligible teachers had prior experience of teaching primary grade levels [1–4] for at least 1 year to avoid teacher acceptability being moderated by inexperience as a teacher, were aged 18 years or above, and had not been convicted of or under active investigation for child maltreatment or misconduct. All 19 teachers who provided permission to their school’s principal to provide their contact information to our team met inclusion criteria and provided informed consent to be in the study. Thirteen teachers completed all study activities. All 6 teachers who left the study did so prior to the collection of quantitative acceptability ratings. Two teachers expressed not being able to provide time for *Tealeaf*. Two teachers cited family reasons. Two teachers were enrolled at a school that felt less comfortable engaging with parents.

*Tealeaf*-trained teachers selected two students each who were in grades 1–4 (5–12 years old) to receive services based on each teacher’s judgment that is grounded in their everyday interactions with students; this method was chosen based on accuracy (as next) and to avoid any additional one-on-one interactions or screenings that may unintentionally identify to others in the classroom or school which children need mental health support. *Tealeaf*-trained teachers have been shown to select children in need of mental health support with moderate accuracy (72% sensitivity and 62% specificity) [36]. Teachers were pragmatically limited to 2 students each based on teacher preferences in an earlier pilot trial [36].

Based on the timing of teacher dropout, verbal assent for participation was obtained in Nepali from 30 students by study staff. An English version of the assent script is in Additional File 1. Written informed consent was obtained from their parent or guardian (termed “caregiver” for this manuscript) for their child’s participation and their own participation in the study. Twenty-six students and their 29 caregivers completed all study activities; 3 children had 2 caregivers each enrolled, one to provide quantitative data and one to provide qualitative data. For qualitative semi-structured interviews, a purposive sample of 7 teachers, 7 children, and their 7 caregivers were chosen as representative of the study sample. To robustly capture potentially different views on acceptability based on gender, teachers were chosen such that a little over half were female, deviating from female representation in the larger study sample.

The research protocol and consent and assent forms were approved by the University of North Carolina at Chapel Hill Institutional Review Board (Study 17–2608) and a Darjeeling-based Ethics Committee.

### Measures

Quantitative and qualitative data, as below, were collected in parallel (i.e., before either was analyzed) due to resource constraints. As in “Analysis” their integration occurred through an explanatory sequential method [32].

#### Quantitative assessments

##### Acceptability

Quantitative measures of acceptability were chosen based on a review of acceptability measures by a panel of experts and research staff with local knowledge. No published surveys assessed parent acceptability of teacher-delivered behavioral and mental health interventions targeting their students. Local research staff considered surveys more generally assessing parent acceptability to be difficult to understand in the Darjeeling context. Thus, the decision was made to assess parent acceptability qualitatively, as below, while proceeding with quantitative assessment of teacher and child acceptability, as next.

##### Intervention rating profile—15 (IRP)

The Intervention-Rating Profile (IRP) assesses teacher acceptability of school-based behavioral and mental health interventions delivered by teachers [37]. Its 15 items are rated on a 6-point Likert-type scale (with 1 indicating strong disagreement and 6 indicating strong agreement). All item scores are added for a total score. Higher scores represent higher levels of acceptability, with IRP authors setting a score of ≥ 70 representing moderate acceptability. The IRP was collected pre-intervention (after teacher training and prior to care delivery, “PRE”) and post- intervention (at the end of the academic year after 6–8 months of care, “POST”). The IRP was translated into Nepali and back translated to English to verify accurate Nepali translation. The IRP took participants 5 min to complete.

##### Children’s intervention rating profile (C-IRP)

The Children Intervention Rating Profile (C-IRP) assesses students’ levels of acceptability of behavioral and mental health interventions delivered by teachers [38]. Seven items are rated on a 6-point Likert rating scale; a score of 1 indicates strong disagreement and 6 indicates strong agreement, with three items reverse scored. Item scores are summed for a total score. Higher total scores indicated higher levels of acceptability, with a score ≥ 24.5 representing acceptability per C-IRP authors [38]. An adapted version of the C-IRP was used to allow students to judge their own participation in *Tealeaf* as the original C-IRP rated students’ levels of acceptability of interventions delivered in vignettes (see Additional File 2) [39]. The C-IRP was collected PRE and POST in Nepali. C-IRP collection at PRE occurred after: (1) children were chosen by teachers for care, (2) informed consent was provided for their participation by caregivers, (3) study staff discussed *Tealeaf* with them and that they were chosen to receive *Tealeaf* care, and (4) they provided verbal assent. The form was translated into Nepali and back-translated to English to ensure accurate translation. The C-IRP took participants 2 min to complete with staff verbal administration.

##### Demographics

All demographics information was collected at PRE. Teacher demographics collected were age, years teaching, years at current school, gender, language(s) spoken, level of education completed, and grade levels taught. To minimize the research burden on caregivers, age, gender, and relationship to the child were collected. Children’s demographics were collected from caregivers and included age, total people living in the household, gender, language(s) spoken, grade level, mental health symptoms as reported by their teacher in the Teacher Report From (as next), and membership in a scheduled caste/tribe (standard terms used in Indian demographic surveys for officially recognized groups of historically disadvantaged peoples by the Government of India and State of West Bengal) [40].

##### Teacher report form (TRF)

The Achenbach System of Empirically Based Assessment (ASEBA) Teacher Report Form (TRF) is a “gold standard” for assessing mental health challenges as reported by teachers [41]. The form includes 113 questions that are scored to produce several clinical scores, with aggregate scores for Total Problem, Internalizing problems, Externalizing problems, and 14 subdomain scores. Raw scores are summed and converted into T-scores. Total problem T-scores from 60 to 63 are classified as “borderline” and ≥ 63 as “clinical”; “borderline” is defined by TRF authors as likely having symptoms that meet diagnostic criteria for a disorder but would be best confirmed by a professional evaluation to minimize false positive screening, where “clinical” is defined as more confidently having symptoms that meet diagnostic criteria for a disorder [41]. Internalizing and Externalizing problem and all subdomain T-scores of 65 to 69 are considered “borderline” and ≥ 70 are “clinical”. The form is available in Nepali and took participants 20 min to complete. For each student, his/her teacher each filled out the TRF at PRE outside of instructional time to protect student confidentiality. The TRF has been validated globally, including in India [42]. However, studies indicate that lower borderline and clinical thresholds than those reported below may be appropriate for the Indian context, leading to a “borderline” score to be the minimum score signaling a need for indicated care [42].

#### Qualitative assessments

With the goal of qualitative description of acceptability, semi-structured interviews (“interviews”) were collected prior to quantitative data analysis with 7 teachers, 7 students and 7 caregivers at POST [43, 44]. Qualitative acceptability aims were modeled after aspects of acceptability described by Proctor and colleagues: acceptability (Aim 1), facilitators of acceptability (Aim 2), conditions required for acceptability (Aim 3), barriers to acceptability (Aim 4), and future directions for acceptability (Aim5) [21]. Interview guides (Additional File 3) were developed iteratively around these aims. The guides were then finalized with the research team and program staff.

Trained research assistants conducted audio-recorded interviews in Nepali and recorded complementary field notes. Interviews were transcribed and translated into English by an independent translator and reviewed by study staff for accuracy.

### Procedures

**Fig. 1:**
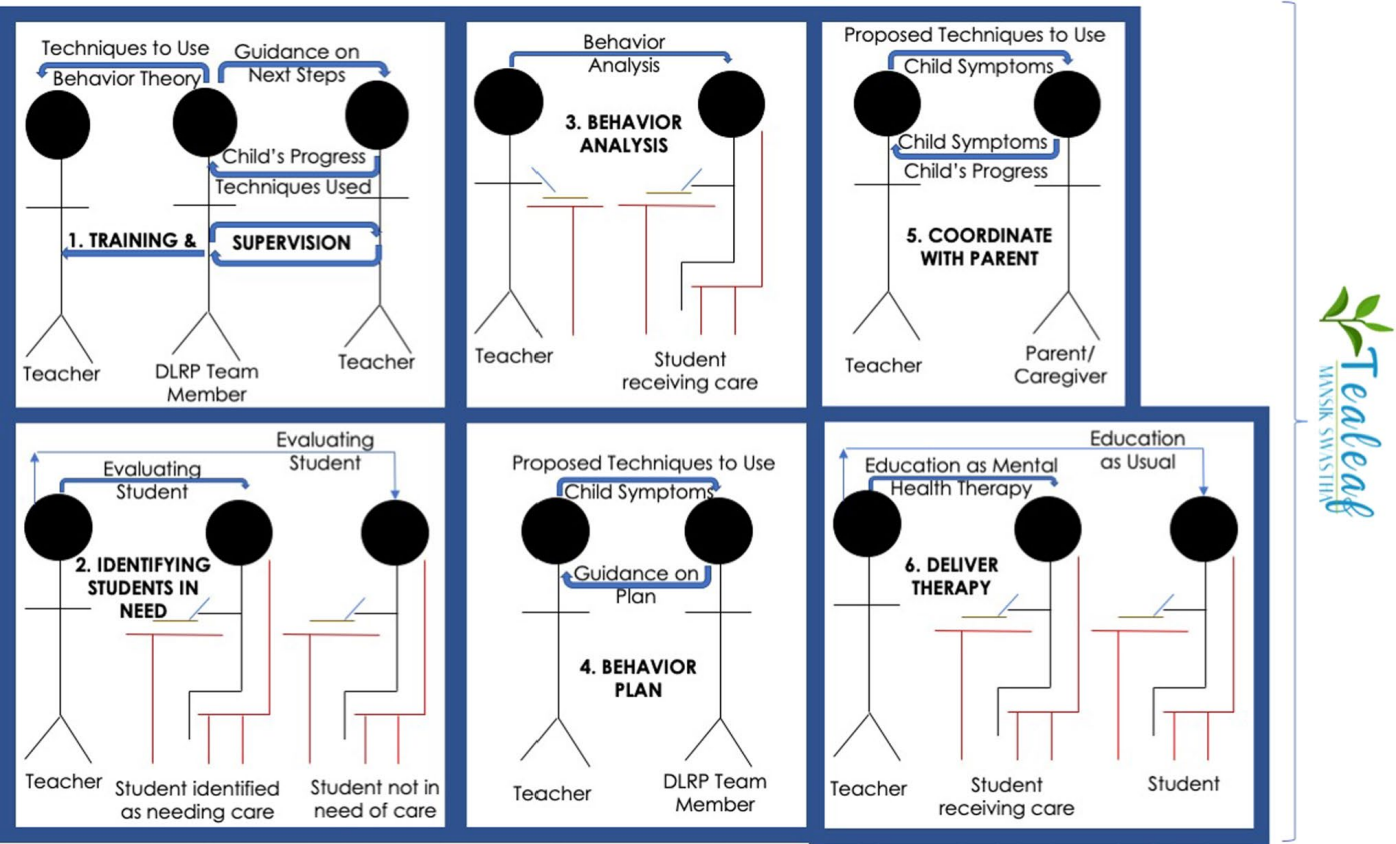
Tealeaf’s 6 components are illustrated here. First, teachers receive training and supervision from study staff. Second, teachers under supervision identify children in need of mental health support in their classrooms. Third, teachers perform a behavior analysis on students to begin to understand targeted behaviors. Fourth, teachers create a behavior plan, choosing therapeutic techniques to use that target behaviors identified in behavior analysis and ones they feel they can implement during the school day. Fifth, teachers coordinate with parents, informing them of their child’s progress at school, learning of their child’s progress at home, and guiding how Tealeaf techniques may be used at home. Finally, teachers deliver therapy during the school day

*Tealeaf* is an intervention that task-shifts to teachers the delivery of evidence-based, non-manualized, transdiagnostic, indicated child mental health care. To address child mental health needs that were considered concerning by the Darjeeling community but for which little care was available (as above), *Tealeaf* was developed by the three authors (CMC, PG, & MM), community partner non-governmental organizations (Darjeeling-based Darjeeling Ladenla Road Prerna and Darjeeling-focused Broadleaf Health and Education Alliance), and their partner LCP schools and community health workers. *Tealeaf* care is unique in that it is non-manualized, allowing teachers to customize the care they deliver, including fitting care tasks into their primary teaching duties. Further, a transdiagnostic approach was chosen to minimize the need for teachers to create differential diagnoses and master individual treatment paradigms for different diagnoses [45]. *Tealeaf* is implemented over a school year and involves six major components: training and supervision, student nomination for care, behavior analysis, behavior plans, one-on-one interaction with students, and engaging caregivers (Table 1 and Fig. 1).

**Table 1:** Core intervention components

Intervention Component	Activity	Description
Professional development & regular supervision	Training	Training: 10-day interactive training for teachers in identification of children with mental health needs, basic functional behavioral assessments and tenets of CBPT, particularly behavior activation, play, and cognitive restructuring Supervision: Twice monthly discussion with and/or observation of the teacher working with the child to provide concrete guidance and technique Case reviews: Monthly case reviews are conducted with a team of local and international mental health experts
Assessment	Student identification	After training, teachers observe their students during typical teaching activities through the lens of identifying who may need mental health support. They choose two students to target for care whom they judge to be in most need based on their observations
Assessment	Behavior analysis	Observations of targeted students through a behavioral lens for key behaviors using the AABC Chart and the Themes of the AABC Chart. Supplemented by collateral from caregivers for further observations from student’s home lives
Tailored instruction; therapeutic interactions & skills practice	Behavior plan	A behavior plan (4Cs) incorporating CBPT and classroom-based therapeutic techniques that target school-specific behaviors (1:1 and during instructional time), to be used daily. Use of plan in student home is highly encouraged
Therapeutic interactions & skills practice	1:1 Student interaction	Per behavior plan students engage in 1:1 interactions with teacher during or outside of class. These interactions include CBPT and relationship-building activities
Therapeutic interactions & skills practice	1:1 Family interaction	With support and guidance from teachers, primary caregivers have roles in behavior analysis, implementation of behavior plans, development of positive parental relationships, and reinforcement of positive behaviors

CBPT, cognitive behavior play therapy; AABC, activating event, automatic thoughts and/or feelings, behavior, & consequence; 4Cs, cause, change, connect, cultivate behavior plan

After a 10-day training delivered by a psychiatric social worker with expertise in youth mental health (component 1 in Fig. 1; Additional File 4), teachers choose two students to work with (component 2 in Fig. 1 and as above in “Participants”), and analyze their behavior by completing basic functional behavioral assessments using 2 decision support tools, the Activating Event, Automatic Thoughts and/or Feelings, Behavior, & Consequence (AABC) Chart and the Themes of the AABC Chart (component 3 in Fig. 1; Additional File 5) [46]. Teachers then develop a targeted response using a behavior plan, the Cause, Change, Connect, and Cultivate (4Cs) Plan (component 4 in Fig. 1; Additional File 5). The 4Cs is akin to behavior plans teachers commonly use in HICs to manage a child’s challenging behavior towards the goal of improved learning, but here with the end goal of improving child mental health [28].

In the 4Cs, teachers select Cognitive Behavior Play Therapy (CBPT)-based therapeutic techniques from: (1) a menu of evidence-based therapeutic options, (2) techniques learned in training that were not on the menu, or (3) techniques they adapted under the guidance of study staff (Additional File 6). CBPT was chosen as *Tealeaf*’s core therapeutic modality as it provided teachers with practical and tangible therapeutic techniques cognitively accessible to children 10 years or under, whereas Cognitive Behavior Therapy (CBT) is effective for those older than 10 years [47, 48].

Through the 4Cs, CBPT tenets can be interwoven into a child’s daily school schedule through the therapeutic choices teachers make, where teachers can choose to deliver care within classroom activities or outside of them in a more traditional 1:1 setting (Additional File 6) [47]. The “dose” of care in *Tealeaf* is each individual therapeutic interaction between a teacher and targeted child throughout the school day, in contrast to traditional models of task-shifted care where “doses” are number and length of 1:1 sessions [7]. This non-manualized modality of therapy, in which teachers deliver care through their existing interactions with students in the classroom using CBPT-based therapeutic techniques only they can use as teachers, is a novel therapy modality named “education as mental health therapy” (Ed-MH), discussed at length in a separate publication from this author group [30].

The remainder of the school year is dedicated to the delivery of therapeutic interactions and skills practice (component 6 in Fig. 1), collaborating with family (component 5 in Fig. 1), and revising the 4Cs based on each child’s progress. From the psychiatric social worker who trained them, teachers receive monthly on-site supervision that is supplemented by as-needed telephone discussions, averaging to twice monthly supervision.

### Data analysis

An explanatory sequential method was pursued to answer the question of whether participants found *Tealeaf* to be acceptable. While data were collected in parallel and seemingly more similar to a concurrent method, the intent of the data analysis was to understand quantitative measures further through qualitative inquiry. Thus, we pursued an explanatory sequential method based on recent mixed methods literature calling for emphasizing the intent of the data analysis rather than the timing of data collection [32]. Quantitative data were first analyzed to gain an understanding of teacher and child acceptability in quantitative terms. To integrate the two forms of data, qualitative data analysis was then undertaken to further explain quantitative results, as well as to understand caregiver acceptability as no quantitative measure was deemed appropriate to measure caregiver acceptability, as above in “Measures”.

#### Quantitative

Descriptive statistics were used to investigate demographic characteristics of all teachers, students, and caregivers enrolled in the study. Demographics of teachers who completed all study activities were compared with those who did not complete all study activities. Demographics of the teachers, students, and parents who completed interviews were compared with those who did not. Independent sample *t* tests were used for continuous variables; Fisher’s exact tests were used for categorical variables.

Mean scores were calculated at PRE and POST time points for the acceptability measurements, C-IRP (Total Acceptability score) and the IRP (Total Acceptability score), and compared using paired sample t-tests (two-tailed). The IRP PRE score was missing for 1 teacher, allowing a PRE to POST comparison for 12 teachers. C-IRP values were compared PRE to POST for children targeted for care who had complete C-IRP and demographics data collected (n = 24). SAS version 9.4 (Cary, NC) was used for all quantitative analyses [49].

#### Qualitative

The interviews from teachers, children, and their parents were analyzed using inductive content analysis with the aim of qualitative description in order to further understand quantitative acceptability scores and caregivers’ perspectives on acceptability [43, 50]. Using ATLAS.ti version 8.4.15, 2019, two independent analysts both coded all of the interviews with a template coding style as per Crabtree and Miller, with one codebook used for all three participant groups [51]. Group consensus was used to resolve discrepancies in coding. Codes were grouped to identify emergent themes alongside key supporting quotations. Results of the analysis were connected to research aims, as in “Measures”, that reflected the aspects of acceptability as described by Proctor and colleagues [21].

## Results

### Demographics

Teachers who completed the study (n = 13) were 76.9% female and ranged in education levels between some primary and finishing graduate/post-graduate; 38.5% had formal education training and 46.2% obtained a teaching certificate (Table 2). Teachers who completed interviews were different in gender proportions from those who did not, intentionally pursued as previously discussed; otherwise, teachers who completed interviewers were not significantly different in demographics from those who did not.

**Table 2:** Teacher demographics and comparisons of teachers who did and did not complete all study activities and those who did and did not complete a qualitative interview

		Comparison of teachers who did and did not complete all study activities			Comparison of teachers who did and did not complete a qualitative interview		
Variable	Enrolled N = 19	Completed N = 13	Did not complete N = 6	T-test p-value^	Completed N = 7	Did not complete N = 12	T-test p-value^
Continuous Variable	Mean (SD)	Mean (SD)	Mean (SD)		Mean (SD)	Mean (SD)	
Age	28.4 (5.8)	29.85 (5.9)	25.17 (4.4)	0.10	27.3 (5.1)	29.0 (6.3)	0.55
Years teaching	6.1 (5.4)	7.4 (5.8)	3.33 (3.4)	0.13	4.6 (6.0)	7.0 (5.1)	0.38
Years at current school	4.4 (4.5)	4.96 (5.0)	3.33 (3.4)	0.48	4.4 (6.2)	4.5 (3.6)	0.95

Categorical variable	N (%)	N (%)	N (%)	Fisher’s exact test p-value^	N (%)	N (%)	Fisher’s exact test p-value^
Female gender	16 (84%)	10 (76.9%)	6 (100%)	0.52	4 (57.1%)	12 (100%)	0.04^
Language							
Nepali	8 (42%)	5 (38.5%)	3 (50.0%)	0.99	2 (28.6%)	6 (50.0%)	0.63
English	6 (32%)	4 (30.8%)	2 (33.3%)	0.99	2 (28.6%)	4 (33.3%)	0.99
Hindi	4 (21%)	2 (15.4%)	2 (33.3%)	0.56	2 (28.6%)	2 (16.7%)	0.60
Level of education				0.54			0.10
Some primary	3 (16%)	2 (15.4%)	1 (16.7%)		1(14.3%)	2 (16.7%)	
Some senior secondary	2 (11%)	2 (15.4%)	0 (0%)		0 (0%)	4 (33.3%)	
Finished senior secondary	4 (21%)	1 (7.7%)	3 (50.0%)		2 (28.6%)	0 (0%)	
Some undergraduate	4 (21%)	3 (23.1%)	1 (16.7%)		1 (14.3%)	3 (25.0%)	
Finished undergraduate	2 (11%)	2 (15.4%)	0 (0%)		1 (14.3%)	1 (8.3%)	
Some graduate/post-graduate	2 (11%)	1 (7.7%)	1 (16.7%)		0 (0%)	2 (16.7%)	
Finished graduate/post-graduate	2 (11%)	2 (15.4%)	0 (0%)		2 (28.6%)	0 (0%)	
Had formal training	5 (26%)	5 (38.5%)	0 (0%)	0.13	2 (28.6%)	3 (25.0%)	0.99
Teaching certificate	7 (37%)	6 (46.2%)	1 (16.7%)	0.33	3 (42.9%)	4 (33.3%)	0.99
Grade level taught							
Nursery/kindergarten	8 (42%)	6 (46.2%)	2 (33.3%)	0.99	3 (42.9%)	5 (41.7%)	0.99
Class I	15 (79%)	10 (76.9%)	5 (83.3%)	0.99	5 (71.4%)	10 (83.3%)	0.60
Class II	14 (74%)	10 (76.9%)	4 (66.7%)	0.99	5 (71.4%)	9 (75.0%)	0.99
Class III	12 (63%)	8 (61.5%)	4 (66.7%)	0.99	5 (71.4%)	7 (58.3%)	0.66
Class IV	12 (63%)	7 (53.9%)	5 (83.3%)	0.33	4 (57.1%)	8 (66.7%)	0.99
Class V	5 (26%)	3 (23.1%)	2 (33.3%)	0.99	2 (28.6%)	3 (25.0%)	0.99
Class VI +	3 (16%)	3 (23.1%)	0 (0%)	0.52	3 (42.9%)	0 (0%)	0.04^^^

^^^Significant if *p* < 0.05

Students (n = 26) were 30.77% female. Those who completed the interview were not different in demographics from those who did not (Table 3). Caregivers enrolled (n = 29) were 82.8% female; 62.1% were mothers (Table 4). Three children had two caregivers each enrolled; only one caregiver per child completed an interview. Those who participated in an interview did not statistically significantly differ from those who did not, though age was not available for the 3 caregivers who completed interviews only. For the three children who had two caregivers each participate in the study, only one caregiver per child (the one who completed an interview) was included in the comparative analysis (n = 26) to allow for balanced representation.

**Table 3:** Child demographics and comparison of children who did and did not complete a qualitative interview

		Comparison of children who did and did not complete a qualitative interview		
Variable	Enrolled (N = 26)	Completed (N = 7)	Did not complete (N = 19)	T-test or fisher’s exact test p-value^a^
Continuous variable	Mean (SD)	Mean (SD)	Mean (SD)	
Age (years)	8.23 (1.7)	8.9 (1.2)	8.0 (1.8)	0.26
Total Household Members	3.8 (1.5)	3.6 (1.1)	3.8 (1.6)	0.69
TRF Total problem score at enrollment^b,c^	62.7 (8.6)	63.7 (10.2)	63.4 (8.4)	0.75

Categorical variable	N (%)	N (%)	N (%)	
Female gender	8 (30.77%)	1 (14.3%)	7 (36.8%)	0.37
Scheduled caste/tribe^d^	7 (26.92%)	1 (14.3%)	6 (31.6%)	0.63
Language				
Nepali	26 (100%)	7 (100%)	19 (100%)	n/c^j^
English	24 (92.31%)	6 (85.7%)	18 (94.7%)	0.47
Hindi	3 (11.54%)	0 (0%)	3 (15.8%)	0.54
Grade				0.07
Class I	4 (15.38%)	0 (0%)	4 (21.1%)	
Class II	6 (23.08%)	0 (0%)	6 (31.6%)	
Class III	9 (34.62%)	5 (71.4%)	4 (21.1%)	
Class IV	5 (19.32%)	1 (14.3%)	4 (21.1%)	
Class VI +	2 (7.69%)	1 (14.3%)	1 (5.2%)	
TRF behavior type				
TRF externalizing at enrollment^c,e^	5 (20.0%)	2 (28.6%)	3 (16.7%)	0.60
TRF internalizing at enrollment^c,e^	13 (52%)	4 (57.1%)	9 (50.0%)	0.99

Baseline Student Mental Health Profile per the ASEBA TRF	
Symptom category	Number of children with positive symptom T-Score (n = 25)^c,f,g^
*Scale Scores*^h,i^	
Total Problem	16 (64.0%)
Internalizing Problems	13 (52.0%)
Externalizing Problems	5 (20.0%)
*Syndrome Scale Scores*^i^	
Anxious/Depressed	10 (40.0%)
Somatic Complaints	4 (16.0%)
Thought Problems	8 (32.0%)
Attention Problems	4 (16.0%)
Rule-Breaking Behavior	2 (8.0%)
Aggressive Behavior	6 (24.0%)
Withdrawn/ Depressed	13 (52.0%)
Social Problems	8 (32.0%)
Any syndrome scale item positive	19 (76.0%)
*Diagnostic and statistical manual (DSM) oriented scales *^*i*^	
Depressive problems	10 (40.0%)
Anxiety problems	13 (52.0%)
Somatic problems	1 (4.0%)
Attention deficit	2 (8.0%)
Oppositional defiant problems	3 (12.0%)
Conduct Problems	4 (16.0%)
Any DSM oriented scale item positive	17 (68.0%)
*Any symptom item positive*^h,i^	
Total Problem, Internalizing, Externalizing, Syndrome, or DSM	20 (80.0%)

^a^Significant if p < 0.05. No comparisons had p < 0.05

^b^T-score ≥ 60 is a borderline score, while > 63 is a clinical score

^c^Calculated based on n = 25 children who had a completed TRF and complete demographic data at baseline

^d^Standard terms used in Indian demographic surveys for officially recognized groups of historically disadvantaged peoples by the Government of India and State of West Bengal

^e^Had at least a of T-score ≥ 65 to meet the cutoff for a borderline score, inclusive of those with scores ≥ 70 (clinical)

^f^A positive score was defined as a borderline or clinical score as per TRF author guidelines

^g^Children receiving care could have positive scores in more than 1 symptom category

^h^Scores under the 60 were considered normal for Total Problem T-scores

^i^Scores under 65 were considered normal for all other T scores

^j^*n/c* not calculable

**Table 4:** Caregiver demographics and comparison of caregivers who did and did not complete a qualitative interview

		Demographic comparison of caregivers who did and did not complete a qualitative interview		
Variable	Enrolled (N = 29)^a^	Completed (N = 7)	Did not complete^b^ (N = 19)	
Continuous variable	Mean (SD)	Mean (SD)	Mean (SD)	T-test p-value^
Age of caregiver^c^	33.6 (11.38)	29.75 (7.1)	33.26 (12.2)	0.59

Categorical variable	N (%)	N (%)	N (%)	Fisher’s exact test p-value^
Female caregiver	24 (82.8%)	5 (71.4%)	17 (89.5%)	0.26
Relationship to child:				0.77
Mother	18 (62.1%)	4 (57.1%)	13 (68.4%)	
Father	4 (13.8%)	1 (14.2%)	2 (10.5%)	
Grandparent	2 (6.9%)	0 (0%)	2 (10.5%)	
Aunt	2 (6.9%)	1 (14.2%)	1 (5.3%)	
Guardian	3 (10.3%)	1 (14.2%)	1 (5.3%)	

^Significant if p < 0.05. No comparisons had p < 0.05

^a^While there were 26 children enrolled, 29 guardians were enrolled as 3 children had two caregivers each enrolled, one who completed the qualitative interview and one who provided the child’s demographic information

^b^For the three children who had two caregivers each enroll, only the caregiver for each who completed qualitative assessment was included in the comparison such that each child only had 1 caregiver included in the comparison. Thus, 26 caregivers of 26 children were included in the comparison

^c^Age not available for three of the caregivers who completed the qualitative assessment

### Quantitative acceptability

Teachers’ IRP scores were, on average, above the moderate acceptability threshold (≥ 70) at PRE (mean = 73.75, standard deviation (SD) = 5.85; n = 12; 75% (n = 9) above acceptability threshold) and POST (mean = 76.92, SD = 5.58; n = 12; 92% (n = 11) above acceptability threshold) (Table 5). Teachers’ scores did not statistically significantly change PRE to POST (mean difference = 3.17; p = 0.1550; 95% Confidence Interval (CI): -1.40, 7.73). Students’ C-IRP scores, on average, showed acceptability of the intervention (score ≥ 24.5) at PRE (mean 29.96, SD = 3.49; n = 24, 96% (n = 23) above acceptability threshold) and POST (mean 27.67, SD = 2.32; n = 24, 96% (n = 23) above acceptability threshold), with the average decline in scores PRE to POST of -2.20 points (95% CI: -3.53, -1.05) being statistically significant.

**Table 5:** Acceptability comparisons

Acceptability Outcome	PRE		POST		POST minus PRE			P-value
	Mean (SD)	Number (%) above acceptability threshold	Mean (SD)	Number (%) above acceptability threshold	Mean (95% CI)	t	df	
IRP (teacher)^a^ n = 12^b^	73.75 (5.85)	9 (75%)	76.92 (5.58)	11 (92%)	3.17 (− 1.40, 7.73)	1.53	11	0.1550
C-IRP (child)^c^ n = 24^d^	29.96 (3.49)	23 (96%)	27.67 (2.32)	23 (96%)	− 2.20 (− 3.53, − 1.05)	− 3.82	23	0.0009*

^a^Acceptability threshold score ≥ 70

^b^Thirteen teachers completed all study activities; IRP PRE was score missing for 1 teacher, allowing PRE to POST comparisons for 12 teachers

^c^Acceptability threshold score ≥ 24.5

^d^Twenty-six students completed all study activities; C-IRP and demographics data were missing for 2 students, allowing PRE to POST comparisons for 24 students

^*^Paired t-test significant if p < 0.05

### Qualitative acceptability

Teachers and caregivers universally expressed acceptability of *Tealeaf* (Aim 1; Table 6). Both groups spoke to the overall program being acceptable, with some explicitly stating wanting it to continue in the future. Teachers further described their acceptability of *Tealeaf’s* individual components. Training and supervision were viewed as venues for learning new, relevant skills, with some of the more experienced teachers expressing these skills were novel and useful even though they had several years of experience. Care delivery was considered to be acceptable, seen as a worthwhile application of new skills learned as efforts were viewed as impactful, as described in Aim 2 below. One teacher stated, *“Through this program I gained new knowledge and skills. Through this program, good changes have come to the children. When I see these changes, I feel happy, and I feel it was worth my time and energy.”*

**Table 6:** Themes and representative quotes from semi-structured interviews with teachers, caregivers, and students

Aim	Theme	Participant Group	Quotation	
Aim 1: Acceptability	Training	Teachers	*“Even though we have had many years of teaching experience, I know that our techniques were sometimes flawed. We learnt a lot of new skills in the training, we learnt how to handle, behave, treat and react to the children.”* *“We are ready to receive and go through more sessions of training too.”*	
	Supervision	Teachers	*“The others (staff) were really nice, they taught us well and they never made us feel that they were teachers. So there was no awkwardness in asking for their help if something was unclear to us or if there was some challenge facing us.”*	
	Delivering care	Teachers	*“Through this program I gained new knowledge and skills, through this program, good changes have come to the children. When I see these changes, I feel happy, and I feel it was worth my time and energy.”* *“We have learnt a lot of things in the training and have gained a lot of knowledge which is more important than the bookish knowledge that anyone can impart. I try to apply on a daily basis all the things that I have learnt.”*	
	Overall program	Teachers Caregivers	*“I hope the program continues in the future.”* *“What I feel is that whatever the program has been doing, it is for the benefit of the children and it’s helped the children and it’s good.”*	
Aim 2: Facilitators of Acceptability	Belief program was impactful	Teachers Caregivers	**Subtheme**	
			Teachers’ beliefs on mental health	*“I would want everyone to understand and be a part of this program because it affects the way you think and react. It really makes you think and reconsider your reactions when the child comes to class without completing their homework or forgets to bring their book.”*
			Teachers’ skills	*“We like the program and we feel happy when we see that we are capable of helping the children.”*
			Child’s academics	*“When I used to teach him, he would not listen to me, he would get distracted, but now he studies very well with concentration and interest. When I see these changes, I feel it is because of the teacher.”*
			Child’s behavior	*“My child was disobedient but through the ideas given by you and the teachers my child has changed and started to become obedient.”*
	Engagement in program	Teachers	*“I am enjoying my time with the kids, getting close to them and learning about child psychology.”*	
	Ability to make adaptations	Teachers	*“I would observe [the selected children] closely but I would never call them separately and conduct special activities because I did not want them to worry and wonder if something was wrong with them.”* *“I make small groups of children who are of the same standard studies wise. And in that group I make sure that one of the selected children gets included. In this way the selected children get extra guidance and the rest of the class gets included too. So no one feels left out and neglected and no one gets to know about the program.”* *“Maybe it’s because of the fact that there is no hard and fast rule regarding the work related to the program so I don’t feel it to be difficult.”*	
	Trust	Caregivers Students	*“The thought that came to my mind was that this program would help my child. There was never any doubt or fear in my mind when I got to know about the program. I took the news as good news because I told myself that the program would help my child in class, especially during studies.”* *“He is very nice and is not strict with us. He loves all of us.”*	
	Communication & relationships	Caregivers	*“It’s very easy to find her and talk to her (teacher). Talking to her was extremely easy and comfortable.”*	
Aim 3: Conditions Required for Acceptability	Understanding of program	Teachers Caregivers	*“Earlier we would treat all the children in the same way in class. We would teach and expect all of the students to get a hang of it. We never considered that the academically weaker students needed more attention. These things were brought into our awareness during the training… When we started doing thing this way, it helped the students.”* *“I have come to understand that this program is here to help the children who have behavioral issues and are weak in their studies. This is a good program and it is here to help our children. They tell us and teaches us how to handle disobedient children and teaches them to cooperate.”*	
	Emphasis on academics over mental health to caregivers	Teachers	*“They were happy when they got to know that their child was chosen for the program because we approached the parents with academics and avoided using the term ‘mental health’ because it is a stigma and still frowned upon. The parents would definitely get anxious and would feel a bit low knowing their child out of all the children in the class had some mental health issue. To avoid this with the parents we told them that we had chosen their child to help them get better at their studies and to help them with their overall development. The caregivers were more than happy to know about this selection and were very supportive.”*	
	Caregiver involvement	Teachers	*“I have had no challenging instances with the parents. The parents come and talk to me about their children. They share their thoughts with me and give me suggestions on how their child can be helped or what else could be done for their child.”*	
	Support from the project team	Teachers	*“Things were a bit easy for us because we did not have to initiate this talk on this topic by ourselves. The teachers from the NGO have organized an awareness program, and it included parents of the students of class 4.”*	
	No observations of negative impact	Caregivers	*“I don’t have much knowledge about this program, but what I have come to understand is that they get to know what knowledge my child has, and whatever issues my child has with studies, this program will help my child handle it*	
Aim 4: Barriers to Acceptability	Lack of time	Teachers	*“Since I am multitasking, I have not been able to give proper time to the children and the program. I am not giving my 100% to the children because it is very difficult for me.”* *“Interviewer (I): Did the teachers always help them complete their work or was it a recent thing that they started to do after learning about the children and the program?* *Teacher (T): This usually does not happen with the children that I have chosen. It’s only during rare moments when they are unable to complete their work or don’t know how to complete their work that they get assistance from the teachers. They don’t always do it for them because all the teachers come with a 40-min lesson plan and they are more focused on completing their lesson plan and have very little time to spare to do these kinds of activities for the two children.”* *“I was unable to give extra time to the two children and could not meet them separately and spend time with them but I was able to give them time when they were working collectively with the entire class. It’s not possible to give those two children extra or special attention in a 45-min class.”*	
	Perceptions of stigma	Teachers Caregivers	*“They obviously did not take it positively. The guardians came to me and said my child isn’t mentally challenged so I don’t think my child should do this program.”* *“I was sure that there were many weak students in his class and wondered why were they not chosen and why was he only chosen for this program.”*	
	Lack of caregiver engagement and understanding	Teachers Caregivers	*“Every parent thinks their child is good; no one wants to consider that their child might be a bit weaker and require help. I just feel they haven’t tried to understand.”* *“Of course my mind and heart had a lot of questions. I would often wonder what would happen to my child. I wondered what would they make my child do, *etc*.”*	
	Not understanding role in program	Students	*“I: Oh, so he taught you in the same old way. Hmmm… Does he care for you and look after you? Does he give you the extra attention or does he treat you like the rest of the class and everyone gets the same treatment?* *Student (S): Everyone equally.”*	
	Distrust of teachers	Students	*“I: Do you feel scared to ask sir for help?* *S: (silence)* *I: huh? Yes or no? Are you scared of him?* *S: Yes* *I: But why do you feel scared?* *S: (long silence)* *I: You can tell me* *S: (silence again)* *I: Are you afraid of him?* *S: Yes* *I: Why are you scared of him?* *S: (Again silence)*	
Aim 5: Future Directions for Acceptability	Program expansion	Teachers Caregivers	*“I can tell you that if the other teachers are trained like the way we are then it is a beneficial program. It will benefit everyone.”* *“It would be fantastic if the program could touch the higher classes of 5, 6, 7.”*	
	Improving caregiver engagement	Teachers	*“I feel we are lagging behind in the area concerning the meeting with the parents of the children. The NGO comes to school and manages to conduct monthly meetings with the teachers, but I have observed that the parents of the selected children get ignored. There was one meeting with the parent right at the beginning of the program but after that there has been no such meeting with the parents.”*	
	Leaving program as is	Caregivers	*“I would like it if the program continued in the coming year as well… because this program brought changes in his behavior. I think there would be more changes if the program continued next year, which would be helpful and beneficial to him.”*	

Most teachers and caregivers expressed that the belief that the program was impactful for students’ behavior and/or academics was a facilitator of acceptability (Aim 2; Table 6). As exemplified in the previous quote, this impact appeared to justify the effort exerted to deliver care, i.e., making the effort acceptable. Further, for teachers, the ability to adapt Ed-MH techniques facilitated acceptability as they could choose techniques as they saw fit rather than adhering to a strict protocol, making the delivery of care seemingly less burdensome and more acceptable. Caregivers’ trust of and communication with teachers facilitated their acceptability as caregivers appeared to use teachers’ endorsement of the program as a proxy for its potential benefit to their child.

A condition required for teacher and caregiver acceptability was having an understanding of the program’s intent (Aim 3; Table 6). Notably, teachers spoke more to the mental health benefits of the program while caregivers emphasized the potential for academic benefits. This discrepancy may stem from teacher communication with caregivers; teachers conditioned acceptability on emphasizing academics over mental health to caregivers and students given their concerns about mental health stigma (Table 6). Teachers also conditioned acceptability on caregiver involvement and support from the project team. Teachers viewed caregivers as being extensions of themselves by supporting child mental health in the home, while support from the project team allowed them to divide care tasks, such as coordinating with parents, and problem-solve on therapy delivery.

Both caregivers and teachers expressed that a perception of stigma was a barrier to acceptability (Aim 4; Table 6). Both were reluctant to identify children, including to the child his or herself, as having a mental health condition for fear of the untoward negative reputation the child could develop in light of mental health being highly stigmatized locally. Both caregivers and teachers also cited a lack of caregiver engagement and understanding as barriers to acceptability (Table 6). Some caregivers did not understand the effects of mental health on daily functioning, such as on academics or behavior, and this may have prevented them from understanding the full benefits of the program. Instead, they may have superficially understood *Tealeaf* to target those with mental health concerns such that the identification of their child as needing mental health support appeared to lead only to stigmatization without clear benefit for such identification. Still, as caregivers universally expressed acceptability of *Tealeaf,* as earlier, this barrier may have played a role in differing levels of acceptability. Some teachers cited a lack of time to deliver the intervention as an acceptability barrier. They reported having school schedules filled with teaching activities, but some reported adapting Ed-MH techniques into whole class activities, for example, to overcome this barrier.

Teachers and caregivers expressed that a future direction (Aim 5; Table 6) could include program continuation in their schools or expansion to either older grades or other schools, implying their acceptability of the program. Some teachers expressed that improving caregiver engagement could enhance future teacher acceptability as teachers viewed caregivers as extensions of themselves within *Tealeaf* in supporting child mental health in the home.

By contrast, interviewed students universally did not know they were receiving care from their teachers (as in “Not understanding role in program” in Aim 4, Table 6). This limited the ability to directly assess their acceptability of teacher-delivered task-shifted mental health care. In one exchange between study staff (labelled as “Interviewer (I)”) and an enrolled child (labeled as “Student (S)”), the child expressed not receiving any additional attention from the teacher.

*“Interviewer (I): Oh, so he taught you in the same old way. Hmmm... Does he care for you and look after you? Does he give you the extra attention or does he treat you like the rest of the class and everyone gets the same treatment?*

*Student (S): Everyone equally.”*

A theme of trust of teachers was expressed by some students (Aim 2; Table 6) while others expressed a distrust of teachers (Aim 4; Table 6). One student stated of his or her teacher, *“he is very nice and is not strict with us. He loves all of us.”* Trust of teachers may be an intermediary in the pathway to student acceptability of teacher-delivered care [27].

## Discussion

This study is the first to explore whether teachers, school-aged students, and their caregivers in an LMIC find teachers delivering task-shifted, transdiagnostic, non-manualized mental health care to their students acceptable, a key factor in care adoption and sustainability. Its findings provide evidence for the acceptability of such care for teachers and children, and interview themes indicate acceptability for the 7 interviewed caregivers. This study is in line with evidence in HICs supporting teacher delivery of indicated mental health care structured similarly to *Tealeaf* and Ed-MH.

Reasons for teacher acceptability are in line with 3 domains determining teacher acceptability of mental health care delivery that Han and Weiss [22] consolidated from the publications of Elliott [23] and Reimers and colleagues [24]. In the first domain of acceptability, the severity of the student’s target mental health concern affects teacher acceptability. Many teachers centered their acceptability around a belief that *Tealeaf* was impactful regarding changing children’s behavior and academics, implying a change in function and symptom severity from a previously more severe state. Accordingly, children receiving care, on average, had improved mental health symptoms outcomes on the TRF, as in a separate publication from this author group [31]. Further, teachers in studies in HICs expressed similar sentiments of acceptability, citing witnessed improved mental health symptoms of students in their classroom, which also implied previously more severe states [16–18].

Notably, these teachers were targeting children with Conduct Disorder and ADHD, diagnoses consider to be “externalizing”, or manifesting in symptoms and behaviors that affect a child’s external world [41]. Children with externalizing symptoms have been more frequently targeted by teachers in HIC studies because of their involvement of the environment versus those with “internalizing” diagnoses, or those that predominantly manifest in the child’s internal psychological world [41, 52–54]. By contrast, baseline student mental health profiles in this study indicate that the children selected for care varied in terms of which categories of diagnoses their struggles were consistent with (Table 3). This finding supports that the transdiagnostic nature of Ed-MH may widen *Tealeaf*-trained teachers’ reach compared to others delivering traditionally-structured care that typically targets single diagnoses [7]. That the care teachers could provide through *Tealeaf* was transdiagnostic and applicable to all categories of diagnoses may have further contributed to teacher acceptability. One teacher noted wanting other teachers to be part of the program as it affected the way he or she thought and reacted, especially towards children who may forgot a book or not complete assignments, implying a reconceptualization of commonly overlooked internalizing symptoms (Table 6) [41, 52–54].

The ability to: (1) choose familiar therapeutic techniques with behavioral or academic actions, (2) make adaptations to these techniques, and (3) not have to adhere to a strict protocol for care, as is the structure of *Tealeaf*, addresses the second domain of teacher acceptability, where the type of care delivered matters [22–24]. By including the choice to use academic and behaviorally based therapeutic techniques, *Tealeaf* was structured to target the improvement of children’s mental health symptoms through methods tangible to, familiar to, and usable by teachers. Such a connection was made through careful choices in the design of therapeutic techniques to target both behavior and mental health. Teacher-delivered efficacious care in HICs targeting Conduct Disorder and ADHD have been similarly flexible and smoothly integrated into teacher workflows, such as teaching of coping skills in a group classroom setting for Conduct Disorder or teacher use of a classroom behavior report card for ADHD [16–18]. Care structured traditionally, as one-on-one sessions, has been mixed in its acceptability across LMIC and HIC settings, potentially due to its structure being less familiar to teachers and more aligned with traditional mental health care, the delivery of which teachers may see as being separate from their teaching duties [10, 22, 25].

Of note, teachers adapted Ed-MH techniques to avoid singling out children receiving care, and such an ability to adapt techniques facilitated their *Tealeaf* and Ed-MH acceptability. Further, a condition of acceptability for both teachers and caregivers was an understanding of *Tealeaf* as a program; tasked to work with caregivers and explain the care as it occurred, teachers took the liberty they were given to explain *Tealeaf* in an understandable and acceptable way to caregivers, ultimately under-emphasizing the mental health benefits of the care and speaking instead to its academic and behavior benefits. These findings highlight that teachers chose to be discrete to optimize the acceptability of *Tealeaf* to caregivers and children, and that having the choice to be discrete (i.e., flexible with care delivery) in turn facilitated their acceptability of *Tealeaf* and Ed-MH.

Their acceptability may have been further facilitated by the care including support and supervision from the project team, a required condition for acceptability. This finding is in line with literature that implicates supervision as crucial to lay counselors’ acceptability of delivering task-shifted care across LMICs and HICs [14, 55–58]. While caregivers were not specifically cited as an additional resource to overcome a lack of time, many teachers discussed the importance of caregiver involvement, whether as a condition for or lack thereof as a barrier to acceptability, similar to mental health professionals’ views [59].

Regarding the third domain, time, some teachers expressed a lack of time to deliver care [22–24]. Teachers’ described their time being consumed by primarily focusing on the knowledge transfer process of education, consistent with other LMIC and many HIC contexts [14, 60]. Some teachers described being unable to give “100%” to the children in *Tealeaf* because of having to balance care delivery and teaching duties. The reported lack of time for some may thus underlie why IRP scores did not change on average PRE to POST. This concern, however, did not appear to significantly affect average teacher acceptability as POST scores remained above the moderate acceptability threshold on average. For some, the additional time needed to deliver care may have been acceptable in return for their stated benefits. One teacher stated,“*through this program I gained new knowledge and skills, through this program, good changes have come to the children. When I see these changes, I feel happy, and I feel it was worth my time and energy*.” For others, they may have adapted Ed-MH techniques to minimize the additional time needed to deliver them, such as utilizing them during instructional time as per one teacher’s description. “*I was unable to give extra time to the two children and could not meet them separately and spend time with them but I was able to give them time when they were working collectively with the entire class. It’s not possible to give those two children extra or special attention in a 45-min class*.”

Themes of caregiver acceptability centered on the school focus and setting of *Tealeaf* and appeared to underlie caregivers’ universal positive acceptability of *Tealeaf*, with sentiments similar to those from a 2018 study exploring caregiver experiences in *Tealeaf* in a separate publication [61]. Such a setting may have been less stigmatizing, similar to findings from studies in HICs showing that caregivers’ acceptability of care was predicated on experiencing less stigma in schools compared to clinic settings and that teacher communication was highly valued [18, 28]. Some caregivers did cite perceptions of stigma as a barrier to acceptability of *Tealeaf,* similar to other studies where stigma was a barrier to caregivers seeking mental health care for their children [62].

Stigma may also underlie why the interviewed students universally did not know they were receiving care. While *Tealeaf* already prioritizes student confidentiality during the identification process, teachers further addressed stigma with children by avoiding singling out students receiving care, an adaptation to *Tealeaf* some chose to pursue that was not a listed therapeutic option in *Tealeaf*. Teachers expressed that delivering *Tealeaf* individually may have made the selected children feel negatively about themselves and labeled them as outsiders to others [63].

It is also possible that stigma may underlie why children’s acceptability scores decreased PRE to POST. No interview themes directly addressed stigma, but exploring themes around trust of teachers may allow for insight. Some students distrusted their “strict” teachers. There is a local cultural expectation that teachers are supposed to be “strict”, and children with mental health struggles are traditionally subject to even more “strictness” from teachers [60]. For students whose C-IRP scores fell PRE to POST, they may have had teachers who were consistently “strict”. Being the recipient of such strictness may have been considered stigmatizing and may have identified them as having more struggles than others. Regardless of whether they knew they were receiving mental health care, students may have used the C-IRP to rate their perceptions of their teachers’ behaviors towards them (i.e., strictness) and how it made them feel (potentially stigmatized), leading potentially to decreasing acceptability scores PRE to POST for these students.

C-IRP scores should be interpreted in the context of interviewed children (n = 7) universally expressing not knowing they were receiving care from their teachers. It is possible that the children not interviewed were more aware of their participation. However, as purposive sampling was pursued, more likely students on average may have been less aware of their participation. As the 7 C-IRP items appear to address program acceptability in the context of a child’s functioning and relationship with their teacher (Additional File 2), children may have rated these aspects on the C-IRP whether or not they were aware of their participation in *Tealeaf.*

### Limitations

With a small, pragmatic sample, results are exploratory, not definitive, and potentially may not occur across a broader sample of teachers, students, and their caregivers. Further, though using statistical tests least sensitive to small sample sizes, the statistical comparisons were conducted with small sample sizes; this potentially could have led to skewed results. Caregivers’ acceptability was not quantitatively measured as no measure was considered appropriate for the situation and context; quantitative caregiver acceptability findings could differ from qualitative findings if collected in the future. We were unable to collect quantitative acceptability ratings and qualitative data from teachers who left the study.

Due to limited resources to interview participants, interviews were limited for teachers (7 of 13), caregivers (7 of 29) and children (7 of 26); qualitative views from the remaining participants may have yielded different findings. In addition, as only a handful of participants from each stakeholder group (teacher, caregiver, and student) participated in the qualitative interviews, saturation was not assessed for, making it uncertain as to whether it was achieved. This limits the generalizability of the qualitative results. Also, interviewees may have wanted to play the “good-participant role” as a demand characteristic and subconsciously or consciously provided positive acceptability answers.

## Conclusion

The positive acceptability teachers, caregivers, and students expressed increases the potential of teacher-delivered, task-shifted child mental health care to be a viable alternative option for care delivery. A teacher’s ability to adapt the intervention and frame it as care that improves behavior and academics without having to emphasize its mental health purpose enabled caregiver acceptability and care delivery without students’ knowledge. Such flexibility is at the heart of *Tealeaf* through having Ed-MH as its therapeutic modality [64]. With teachers being able to disguise care as an educational intervention, Ed-MH may be a feasible option for delivering care where mental health stigma is widespread.

Given the acceptability findings presented here and previous findings showing that teachers can identify children with mental health needs with moderate accuracy [36], a logical next step is to definitively assess the ability of Ed-MH and *Tealeaf* to improve child mental health outcomes. Should evidence support that Ed-MH within *Tealeaf* improves child mental health outcomes, teachers may prove to be practical, ubiquitous, and experienced human resources who can take significant steps to bridge the wide child mental health care gap in LMICs through delivering education repurposed for mental health, even and especially in settings with high levels of mental health stigma.

## Supplementary Information

Below is the link to the electronic supplementary material.

Supplementary file1 (DOCX 23 KB)

Supplementary file2 (DOCX 3542 KB)

Supplementary file3 (DOCX 21 KB)

Supplementary file4 (DOCX 19 KB)

Supplementary file5 (DOCX 525 KB)

Supplementary file6 (DOCX 21 KB)

## Data Availability

The datasets generated and/or analyzed during the current study are not publicly available due to the connectedness of the Darjeeling community, the relatively small sample size of teachers, caregivers, and students included where families may be able to connect which children they know received services, and with mental health continuing to be stigmatized in the Darjeeling area. Participants did not agree to share their data publicly. Requests for data will be reviewed case-by-case by the corresponding authors upon reasonable request.
