# Supplementary material for: Teacher, caregiver, and student acceptability of teachers delivering task-shifted mental health care to students in Darjeeling, India: a mixed methods pilot study
Source: Discov Ment Health. 2022 Oct 31;2(1):21. doi: 10.1007/s44192-022-00024-z (PMC9622553; doi:10.1007/s44192-022-00024-z)
Supplement: Supplementary file 1 — Supplementary file1 (DOCX 23 KB) [file 44192_2022_24_MOESM1_ESM.docx]

**University of North Carolina at Chapel Hill
Assent to Participate in a Research Study
Minor Subjects (7-14 yrs)**
________________________________________________________________________
**Consent Form Version Date:** December 14, 2018
**IRB Study #** 17-2608
**Title of Study:** Task-Shifting Children's Mental Health Treatment to Classroom Teachers.
**Person in charge of study:** **intentionally blank for peer review of publication
**Where they work at UNC-Chapel Hill:** Psychiatry
**Other people working on this study:** **intentionally blank for peer review of publication
**Study Contact Telephone Number**: **intentionally blank for peer review of publication
**Study Contact Email**: **intentionally blank for peer review of publication

The people named above are doing a research study.

**These are some things we want you to know about research studies:**
Your parent needs to give permission for you to be in this study. You do not have to be in this study if you don’t want to, even if your parent has already given permission.

You may stop being in the study at any time. If you decide to stop, no one will be angry or upset with you.

Sometimes good things happen to people who take part in studies, and sometimes things happen that they may not like. We will tell you more about these things below.

**Why are they doing this research study?**
The researchers are interested in helping improve the health and learning opportunities of students. This year, they will be running a program to help train teachers to support and help students.

The reason for doing this research is to determine whether this program is interesting and helpful.

**Why are you being asked to be in this research study?**
You are being asked to participate in this research study because your teacher is participating in the program. Your participation is voluntary, meaning that you do not have to participate. You can decide to stop being in the program at any time without getting into trouble.

This is a study you have been a part of for the last year. The researchers are extending the study an additional year to have your teacher continue to work with you.

**How many people will take part in this study?**
If you decide to be in this study, you will be one of about 400 people in this research study.

**What will happen during this study?**
If you agree to be in the program, your teacher will work with you during this school year.

Like last year, your teacher may do special activities with you. Other students may not do these activities. You may also have special rules and rewards that are different from other students. Your teacher will spend time with you talking about your thoughts and feelings. Your teacher will also talk with your parents about the activities he/she is doing with you.

If you agree to participate in the program you will also be part of the study again, this time for this second year. This means, the researchers will collect information that may be about you. When the researchers tell other people about the study, they will not use your name, and no one will be able to tell we are talking about you.

The study will take place at your school and will last for the rest of the school year for a second year.

**Who will be told the things we learn about you in this study?**
The research team will have access to the information collected. Your teacher will also have access to information that you share with them and your teacher may choose to share this information with your parents if they feel it is important.

The researchers will not tell anyone what you tell us without your permission unless there is something that could be dangerous to you or someone else.

**What are the good things that might happen?**
Research is designed to benefit society by gaining new knowledge. The benefits to you from being in this study may be that you feel happier, learn better, and behavior better in school.

**What are the bad things that might happen?**
Sometimes things happen to people in research studies that may make them feel bad. These are called “risks.” The risks of this study include that you might feel embarrassed, upset, or emotionally uncomfortable at times. Some people cry or get upset when they discuss painful feelings, emotions, and experiences. This may happen to you.

Not all of these things may happen to you. None of them may happen or things may happen that the researchers don’t know about. You should report any problems to the researcher

**Will you get any money or gifts for being in this research study?**
You will not be receiving any money or gifts for taking part in this study.

**Who should you ask if you have any questions?**
If you have questions you should ask the people listed on the first page of this form. If you have other questions, complaints or concerns about your rights while you are in this research study you may contact the Institutional Review Board at 919-966-3113 or by email to IRB_subjects@unc.edu.

If you sign your name below, it means that you agree to take part in this research study.

|  |  |
| --- | --- |
| ______________________________________________________ Sign your name here if you want to be in the study | ____________________ Date |
| ______________________________________________________ Print your name here if you want to be in the study |  |

|  |  |
| --- | --- |
| ______________________________________________________ Signature of Research Team Member Obtaining Assent | ____________________ Date |
| ______________________________________________________ Printed Name of Research Team Member Obtaining Assent |  |
