# Supplementary figures and images for: Teacher, caregiver, and student acceptability of teachers delivering task-shifted mental health care to students in Darjeeling, India: a mixed methods pilot study
Source: Discov Ment Health. 2022 Oct 31;2(1):21. doi: 10.1007/s44192-022-00024-z (PMC9622553; doi:10.1007/s44192-022-00024-z)

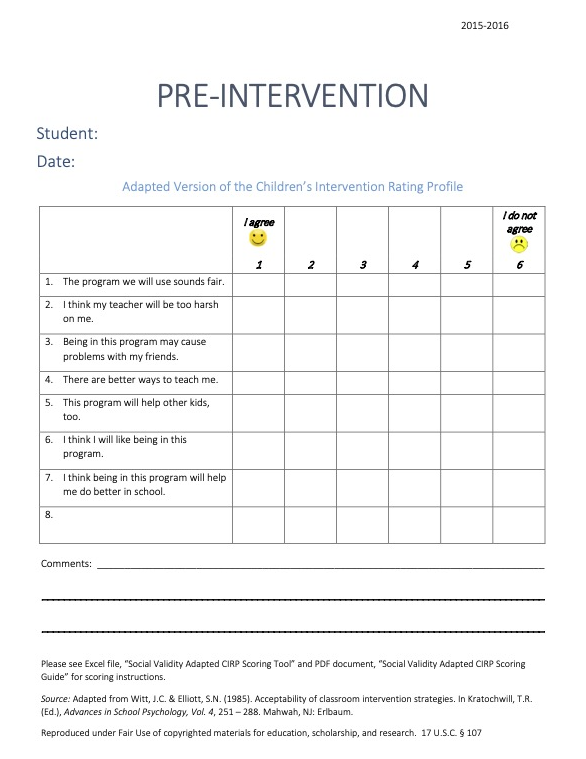

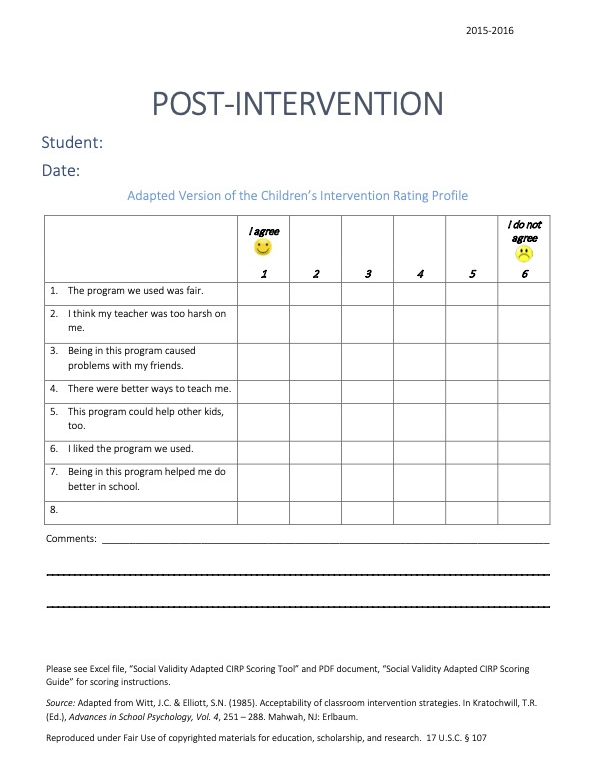

Supplement: Supplementary file 2 — Supplementary file2 (DOCX 3542 KB) [file 44192_2022_24_MOESM2_ESM.docx]
