# Supplementary material for: Teacher, caregiver, and student acceptability of teachers delivering task-shifted mental health care to students in Darjeeling, India: a mixed methods pilot study
Source: Discov Ment Health. 2022 Oct 31;2(1):21. doi: 10.1007/s44192-022-00024-z (PMC9622553; doi:10.1007/s44192-022-00024-z)
Supplement: Supplementary file 3 — Supplementary file3 (DOCX 21 KB) [file 44192_2022_24_MOESM3_ESM.docx]

**Classroom Teachers Semi-Structured Interview Guide**

***I am [name of research staff] from Darjeeling Prerna. Today we are conducting a participatory assessment of the child mental health program. This research will be used to help us understand the benefits and challenges of the program. We will use this information as part of our study to understand how the program worked and how we can improve it in the future.***

***As a reminder, your participation in this interview is voluntary. We can stop at any time. If any of the interview questions make you uncomfortable or upset, you can choose not to answer.***

***We will be audio recording this interview so that we can capture everything that you share with us. The tapes will be stored in our office in a locked cabinet and we will destroy them upon completion of the study. Everything that you share with us will be kept confidential.***

***We greatly appreciate you taking the time to share your thoughts and experiences with us!***

***I’d like to start by asking you some questions about the benefits of the child mental health program.***

1. Do you think selected students benefitted from this intervention? (*Probe: why/why not? Probe for specific examples)*
2. Did you notice any negative impacts on students selected for intervention? (*Probe for specific examples)*
3. Do you think other students in your class who were not selected for the intervention benefitted from the program? (*Probe: why/why not? Probe for specific examples)*
4. Did you feel that the impact of the program (on you and your students) was worth the time and energy you devoted to it? (*Probe: why/why not?)*

***Now I’d like to ask you about the challenges of this program.***

1. Do you think this intervention took too much time, too little time, or just enough time? (*Probe: why/why not?)*
2. Was it more difficult to meet the needs of other students in the class due to the extra time dedicated to students selected for the program? (*Probe: why/why not?)*
3. Would you be willing to deliver this intervention along with your regular job responsibilities? (*Probe: why/why not?)*
4. Should this work be financially compensated? (*Probe: if so, what would be a rough estimate of an appropriate compensation?)*

***Now I’d like to ask you about social dynamics surrounding the program.***

1. Did parents understand that their children were selected for the program due to behavioral/mental health concerns? (*Probe for specific examples)*
2. Did any parents seem uncomfortable or upset that their children were selected for the program? (*Probe for specific examples)*
3. Did the other students in the class seem aware that selected students were receiving the program? (*Probe for specific examples)*
4. Did the students receiving the program appear to have problems with their peers (for example, teasing or bullying) due to their involvement in the program? (*Probe for specific examples)*
5. Do you think that other teachers in your school treated students receiving the program differently because of their involvement in the program? (*Probe for specific examples)*

***Now I’d like to ask you about the future of the program***

1. Would you participate in the program again? (*Probe: why/why not?)*
2. Do you think other teachers who participated in the program this year would be willing to do so again? (*Probe: why/why not?*)
3. Do you think other teachers in your school would be willing to participate in the program next year? (*Probe: why/why not?)*
4. Do you have any other thoughts or suggestions for us regarding the program and how we can make it better in the future?

***Thank you for everything that you have done during this study and program. We greatly appreciate your participation! Please feel free to reach out to our team if you have any thoughts, questions, or concerns.***

***Thank you.***

**Parents & Caregivers Semi-Structured Interview Guide**

***I am [name of research staff] from Darjeeling Prerna. Today we are conducting a participatory assessment of the child behavioral health program. This research will be used to help us understand the benefits and challenges of the program. We will use this information as part of our study to understand how the program worked and how we can improve it in the future.***

***As a reminder, your participation in this interview is voluntary. We can stop at any time. If any of the interview questions make you uncomfortable or upset, you can choose not to answer.***

***We will be audio recording this interview so that we can capture everything that you share with us. The tapes will be stored in our office in a locked cabinet and we will destroy them upon completion of the study. Everything that you share with us will be kept confidential.***

***We greatly appreciate you taking the time to share your thoughts and experiences with us!***

1. **Introduction**

***I’d like to ask you some general questions about the child behavioral health program.***

1. Can you describe for me your understanding of the purpose of the program?
2. In general, how do you feel the program went for your child?
3. Did your child benefit from this program? (*Probe for specific examples)*
4. What challenges did you experience with the program? (*Probe for specific examples)*
5. Do you feel like the program was overall successful or unsuccessful in supporting your child? (*Probe: why/why not?)*

***Now I’d like to ask you more about your child’s involvement in the program.***

1. How did the teacher support your child? (*Probe for specific examples)*
2. Did this support help your child? (*Probe: why/why not? Probe for specific examples)*
3. Do you feel that the teachers were appropriately trained and qualified to provide this support? (*Probe: why/why not? Probe for specific examples).*
4. Did your child experience any negative consequences as a result of the support? (*Probe for specific examples)*

***Now I’d like to ask you about social dynamics surrounding the program.***

1. What was it like for you to have your child identified as requiring extra mental health/behavioral support? (*Probe for specific examples*)
2. Do you feel like your child needed this program and additional support? (*Probe: why/why not?)*
3. What did other members of your family think about your child’s involvement in the program? (*Probe for specific examples)*
4. Were other students and parents aware that your child was part of the program? *(Probe: If so, did students and parents treat your child differently because of that knowledge?)*
5. Did your child’s involvement in the program impact how others in the community viewed your family? (*Probe for specific examples)*
6. Did this program result in any negative consequences for you and your family? (*Probe for specific examples)*
7. Do you feel as though you view your child differently as a result of this program? *(Probe: why/why not?*)

***I’d like to ask you some questions about the future of the program.***

1. Would you like your child to continue to receive support from this program in the future? (*Probe: why/why not?)*
3. Do you have any thoughts or suggestions for us regarding the program and how we can make it better in the future?

***Thank you for everything that you have done during this study and program. We greatly appreciate your and your child’s participation! Please feel free to reach out to our team if you have any thoughts, questions, or concerns.***

***Thank you.***

**Student Semi-Structured Interview Guide**

***I am [name of research staff] from Darjeeling Prerna. Today we would like to talk to you about the extra support you received from your classroom teacher this year. This research will be used to help us understand the benefits and challenges of the program that provided that extra support. We will use this information as part of our study to understand how the program worked and how we can improve it in the future.***

***As a reminder, your participation in this interview is voluntary. We can stop at any time. If any of the interview questions make you uncomfortable or upset, you can choose not to answer.***

***We will be audio recording this interview so that we can capture everything that you share with us. The tapes will be stored in our office in a locked cabinet and we will destroy them upon completion of the study. Everything that you share with us will be kept confidential.***

***We greatly appreciate you taking the time to share your thoughts and experiences with us!***

***I’d like to start by asking you some questions about the support you received through this program.***

1. What was the extra support you received from your teacher like? (*Probe for specific examples)*
2. What activities did you do with your teacher that other kids did not do? (*Probe for specific examples)*
3. What did you like about these activities? (*Probe for specific examples)*
4. What did you not like about these activities? (*Probe for specific examples)*
5. Did you think the extra attention and activities helped you succeed in school? (*Probe: why/why not?)*
6. Did you like going to school more because of the extra support? (*Probe: why/why not?)*

***Now I’d like to ask how your participation in this program affected you and your relationships with other kids in your class.***

1. How did you feel about receiving extra support from your teacher? (*Probe for specific examples)*
2. Do you know why you received extra support?
3. Did you feel as though other kids treated you differently because you were receiving extra support? (*Probe for specific examples)*

***Now I’d like to ask you about doing this program again in the future.***

1. Would you like to receive this extra support and attention again next year? (*Probe: why/why not?)*
