# Supplementary material for: Teacher, caregiver, and student acceptability of teachers delivering task-shifted mental health care to students in Darjeeling, India: a mixed methods pilot study
Source: Discov Ment Health. 2022 Oct 31;2(1):21. doi: 10.1007/s44192-022-00024-z (PMC9622553; doi:10.1007/s44192-022-00024-z)
Supplement: Supplementary file 4 — Supplementary file4 (DOCX 19 KB) [file 44192_2022_24_MOESM4_ESM.docx]

# **Additional File 4.** Training Outline

*10-day Training Curriculum Outline*

10-day training focuses on identifying students with mental health concerns, completing behavior analyses, creating and adhering to a behavior plan and delivering CBPT

- **Orientation to the Training and Intervention** (Day 1, ½ day)
  - **Pre-training summative assessment**
- **Module 1: Understanding Behavior** (Day 2)
  - **Introduction to Behavior**
  - **Behavior has specific functions or motives and can have more than one function**
  - **Behavior can be productive or disruptive**
  - **Behavior is triggered, leading to patterns of behavior**
  - **Poor behavior may indicate a missing skill**
  - **Poor behavior is often atypical behavior**
  - **Building relationships: the key to behavior change**
  - **Living Example**
- **Module 1.5: Approaching Your Students and Community** (Day 3)
  - **Why do the details of communication matter?**
  - **Why are behavioral issues so sensitive?**
  - **Communicating with students**
  - **Communicating with families**
  - **Communicating with colleagues**
  - **The power of relationships**
  - **Self-awareness: recognizing your own biases while supporting the student**
- **Module 2: The 4Cs Plan** (Day 4 and 5)
  - **What are behavior plans?**
  - **What is the 4Cs plan?**
  - **4Cs: Cause**
  - **4Cs: Change**
  - **Teaching new skills**
    - **Cognitive Behavioral Play Therapy (CBPT)**
  - **4Cs: Connect**
  - **4Cs: Cultivate**
  - **Choosing the Right 4Cs Plan**
- **Module 3: Working with Nervous Children** (Day 6)
  - **Introduction to Anxiety**
  - **4Cs: Cause for anxious students**
  - **4Cs: Change for anxious students**
    - **CBPT**
  - **4Cs: Connect for anxious students**
  - **4Cs: Cultivate for anxious students**
  - **Creating a 4Cs plan for a student with anxiety**
- **Module 4: Working with Disagreeable Children** (Day 7)
  - **What does it mean to be “disagreeable”?**
  - **4Cs: Cause for students with disagreeable behavior**
  - **4Cs: Change for disagreeable students**
    - **CBPT**
  - **4Cs: Connect for disagreeable students**
  - **4Cs: Cultivate for disagreeable students**
  - **Creating a 4Cs plan for a disagreeable student**
- **Module 5: Working with Withdrawn Children** (Day 8)
  - **What does it mean to be “withdrawn”?**
  - **4Cs: Cause for students with withdrawn behavior**
  - **4Cs: Change for withdrawn students**
    - **CBPT**
  - **4Cs: Connect for withdrawn students**
  - **4Cs: Cultivate for withdrawn students**
  - **Creating a 4Cs plan for a withdrawn student**
- **Review of Study Protocols** (Day 9)
  - **Child Abuse**
  - **Suicidal Ideation or Attempt**
- **Conclusion** (Day 10, ½ day)
  - **Feedback from participants about training**
  - **Post-training summative assessment**
