# Supplementary material for: Teacher, caregiver, and student acceptability of teachers delivering task-shifted mental health care to students in Darjeeling, India: a mixed methods pilot study
Source: Discov Ment Health. 2022 Oct 31;2(1):21. doi: 10.1007/s44192-022-00024-z (PMC9622553; doi:10.1007/s44192-022-00024-z)
Supplement: Supplementary file 5 — Supplementary file5 (DOCX 525 KB) [file 44192_2022_24_MOESM5_ESM.docx]

# **Additional File 5**. AABC Chart: Cause Analysis Chart

**
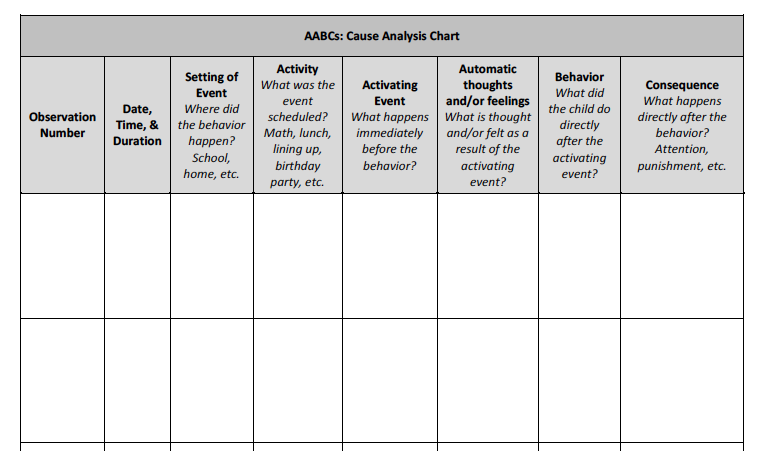
**

**
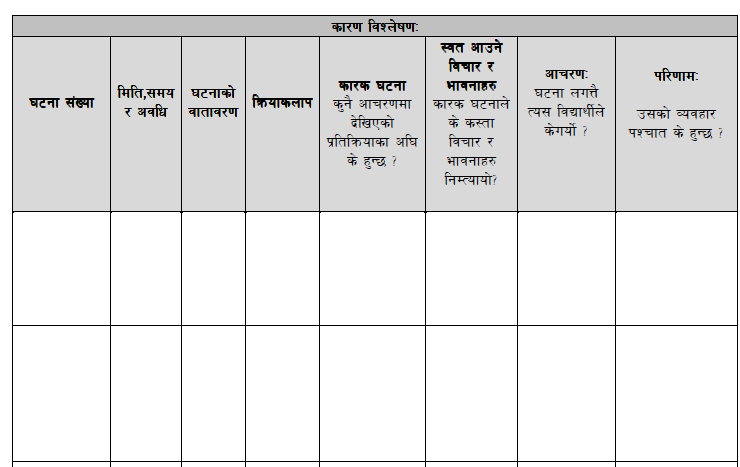
**

**Additional File 5**. Themes of AABC Chart

| **Themes of AABC Chart** | | |
| --- | --- | --- |
| **Date, Time, & Duration** | 1. Common days of the week 2. Common time of day when the Behavior occurs (specify morning, afternoon, or evening) |  |
| **Setting of Event** | 1. School 2. Home 3. Both 4. If other, specify |  |
| **Activity** | 1. Specific subject (specify subject) 2. Unstructured time (lunch, recess, break time, or specify if other) 3. Transitions (specify from what/where and to what/where is the transition) 4. If other, specify |  |
| **Activating Event** *What happens immediately before the behavior?* | 1. Peer interaction (specify socializing, being alone, or specify if other) 2. Adult interaction (specify school staff, family, or specify if other) 3. Involving a challenging task for the student (specify task) 4. If other, specify |  |
| **Automatic thoughts and/or feelings** *What is thought and/or felt as a result of the activating event?* | 1. Thought types: self-image or others' images, one's or others' capabilities, one's self-worth or others' worth, worry for self or other, injustice, betrayal 2. Feelings types: angry, frustrated, sad, happy, scared 3. If other, specify 4. Core beliefs and evidence to support |  |
| **Behavior** *What did the child do directly after the activating event?* | 1. Physical behavior (specify aggression (specify if towards person, animal, property, or specify if other), yelling, crying, sleeping, silent, or specify if other) 2. Mood (specify happy, sad, angry, worried, blunted, or specify if other) 3. If other, specify |  |
| **Consequence** *What happens directly after the behavior?* | 1. Attention (specify positive or negative) 2. Escape 3. Tangible 4. Sensory 5. If other, specify |  |

**
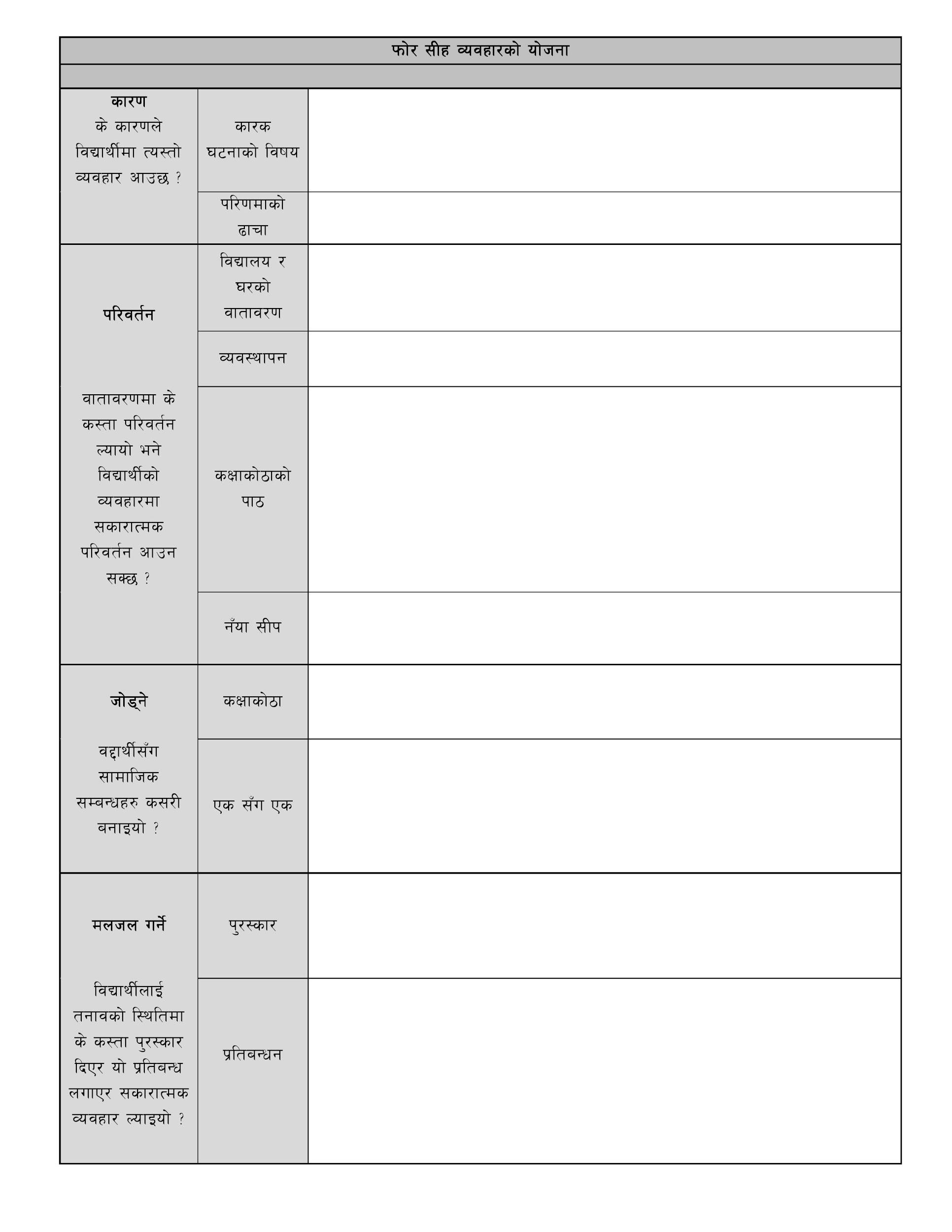

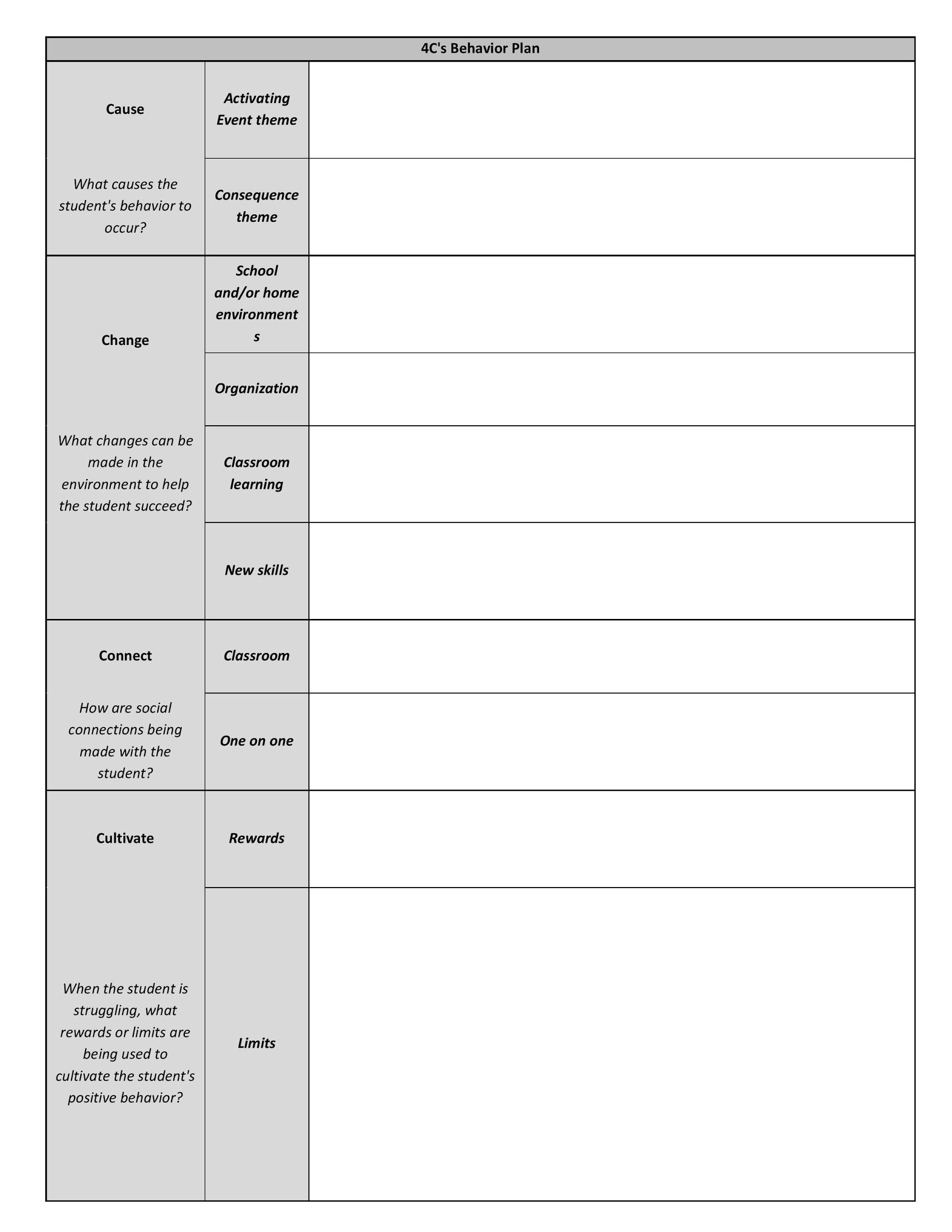
 Additional File 5**. 4Cs Behavior Plan
