# Supplementary material for: Teacher, caregiver, and student acceptability of teachers delivering task-shifted mental health care to students in Darjeeling, India: a mixed methods pilot study
Source: Discov Ment Health. 2022 Oct 31;2(1):21. doi: 10.1007/s44192-022-00024-z (PMC9622553; doi:10.1007/s44192-022-00024-z)
Supplement: Supplementary file 6 — Supplementary file6 (DOCX 21 KB) [file 44192_2022_24_MOESM6_ESM.docx]

**Additional File 6**. Menus of Therapeutic Techniques, per Behavior Category

|  | **Nervous** | **Disagreeable** | **Withdrawn** |
| --- | --- | --- | --- |
| **Cause** | - AABC Chart - Themes of the AABC Chart   - Attention   - Escape   - Tangible   - Sensory | - AABC Chart - Themes of the AABC Chart   - Attention   - Escape   - Tangible   - Sensory | - AABC Chart - Themes of the AABC Chart   - Attention   - Escape   - Tangible   - Sensory |
| **Change** | - School environment   - Schedule preventative breaks   - Small group settings - Organization/planning   - Read the room   - Visual timers   - Avoid timed tests and games   - Visual schedules   - Schedule time to organize belongings - Classroom learning   - Pre-determined visual cues for writing   - Use computers or mobile phones for word processing when subject is not penmanship   - Spelling accommodations when subject is not spelling   - Checklist of weakness and strategies to use in the moment   - Reduce expected completed work   - Preview work for the day for 5 minutes before the day starts   - Reduce, accommodate, or eliminate homework - New skills   - Emotion recognition     - 1:1 when calm     - In the moment     - Emotion color chart     - Emotion thermometer   - Linking emotion to behavior     - CBPT or CBT   - Self – regulation     - Calming box     - Physical coping strategies     - Imagery     - Music     - Coloring   - Targeting automatic thoughts and feelings     - Thought stopping (CBT)     - Power cards   - Challenging core beliefs   - Self-monitoring | - School environment   - Schedule preventative breaks   - Daily schedule which alternates preferred and non-preferred activities   - Alternate lunch and/or recess   - Purpose during transitions   - Give warnings for end of activity and then start of next activity   - Distraction during downtime - Organization/planning   - Visual timers   - Visual schedules and preview them - Classroom learning   - Embed choice into the learning   - Reduce, accommodate, or eliminate homework - New skills   - Emotion recognition   - Self – regulation     - Calming box     - Physical coping strategies     - Imagery     - Music     - Coloring   - Targeting automatic thoughts and feelings     - Power cards   - Challenging core beliefs     - Being Responsible worksheet | - School environment   - Calming box   - Buddy system at recess   - Recess plan   - Alternate lunch and/or recess - Organization/planning   - Checklist   - Visual schedules   - Visual timer   - Present only a few problems at a time - Classroom learning   - Make lesson multi-sensory   - Classroom assignments on topics interesting to the withdrawn student   - Reduce, accommodate, or eliminate homework   - Extra time to complete exams and extra day(s) to complete homework assignments. - New skills   - Emotion recognition     - Color chart with added self-regulation checklist     - Daily self-reflection sheet   - Self – regulation     - Calming box     - Physical coping strategies     - Imagery     - Music     - Coloring     - Desk skills     - Heavy physical activity   - Reframe negative thoughts     - Thought stopping     - Balancing negative thoughts/feelings with positive ones     - Power cards   - Ask for a break appropriately |
| **Connect** | - 1:1 tasks - Baseline of child’s behavior - Regular check-ins to prompt regulation strategy - Use simple, concise language - Make empowering statements when distressed - Build student’s self-esteem   - Non-contingent reinforcement   - Leadership roles | - 1:1 tasks - Baseline of one’s feelings of child’s behavior - Using gentle, specific language when giving demands - Reinforce appropriate behavior   - Continuous positive attention   - Intermittent reinforcement   - Non-contingent reinforcement - Lighten up the moment - Collaborate with the student’s family | - 1:1 tasks - Baseline of one’s feelings on child’s behavior - Narrate your time with the student - Narrate the student’s time with others - Student leadership opportunities - Collaborate with the student’s family |
| **Cultivate** | - Use breaks as rewards - Non-preferred activity for 5 minutes before a 20 minute break - Rewards to reinforce use of self-soothing strategy - Positive verbal reinforcement in the moment of anxiety to reinforce self-soothing - Have student keep track of behaviors and strategies | - Acknowledge only positive behavior - Non-preferred activity for 5 minutes before a 20 minute break - Validate the child’s feelings and then provide a choice - Use incremental consequences - Label the disagreement and encourage positive behavior - Avoid prolonged discussions | - Provide positive feedback   - Continuous positive attention   - Non-contingent reinforcement - Avoid giving negative attention   - Reframe negative thoughts in the moment   - Neutral and quick response |
